# *Bordetella bronchiseptica *pneumonia in a man with acquired immunodeficiency syndrome: a case report

**DOI:** 10.1186/1752-1947-3-76

**Published:** 2009-10-15

**Authors:** Michal Galeziok, Ingram Roberts, Jo-Anne Passalacqua

**Affiliations:** 1 Department of Medicine, St. Vincent's Medical Center, 2800 Main Street, Bridgeport CT 06606, USA

## Abstract

**Introduction:**

*Bordetella bronchiseptica *can be a cause of virulent pneumonia in humans with impaired immune systems. Few cases have been reported in the medical literature where *Bordetella bronchiseptica *has been the only pathogen isolated during a course of interstitial pneumonia.

**Case presentation:**

A 42-year-old African-American man with human immunodeficiency virus presented with pulmonary symptoms that mimicked *Pneumocystis jiroveci *pneumonia. A sputum culture grew *Bordetella brochiseptica*, a common respiratory commensal of wild and domestic animals, rarely implicated in human infections.

**Conclusion:**

*Bordetella bronchiseptica *should be added to the differential list of pathogens which can affect people with human immunodeficiency virus and pulmonary symptoms. Sputum culture, as well as history of animal exposure, in these patients is advised.

## Introduction

*Bordetella bronchiseptica *is a common pathogen in the respiratory tract of many wild and domestic animals, but it is rarely found in humans [1]. This pathogen is believed to be transmitted from dogs with tracheobronchitis [2]. *Bordetella bronchiseptica *is considered an etiologic agent of upper respiratory tract infections, pneumonitis, endocarditis, peritonitis, meningitis, sepsis and recurrent bacteraemia in people with immunological abnormalities [3]. In human immunodeficiency virus and/or acquired immunodeficiency syndrome (HIV/AIDS) patients, *Bordetella bronchiseptica *can cause an interstitial pneumonia which can resemble pneumocystis pneumonia.

## Case presentation

A 42-year-old African-American man with HIV/AIDS, not taking any medications including HAART and pneumocystis jiroveci prophylaxis since January 2007, presented in August 2007 with complaints of progressing shortness of breath, right-sided pleuritic chest pain, non-productive cough, and low-grade fever for last several weeks. He was diagnosed with HIV initially in 1999, during hospitalization for community-acquired pneumonia. The patient also had a history of facial herpes simplex virus type 2 (HSV-2) which has been recurrent and a history of pneumocystis pneumonia in June 2005. Previous laboratory studies from November 2006 revealed a CD4+ lymphocyte count of 20 and viral load HIV RNA of 65,833 copies/ml. He was a social drinker and had never used illicit drugs or tobacco products. HIV was presumed to be acquired sexually.

On initial assessment in the emergency department, he was cachectic but was in no acute distress. He had a fever of 38°C, blood pressure of 92/60 mmHg and regular pulse of 85 beats per minute. He was breathing at 20 breaths per minute. Oxygen saturation was 95% on room air, which decreased to 90% during ambulation. There were no oral lesions. His neck was supple. Examination of the lungs revealed bilateral expiratory wheezes and rare rhonchi. Cardiac examination demonstrated normal first sound, second sound with a regular rhythm and no murmurs. His abdomen was soft, nontender, nondistended with normoactive bowel sounds. His extremities were warm and his skin was dry with multiple small herpetic ulcers on the left ear and the left side of the face.

**Figure 1:**
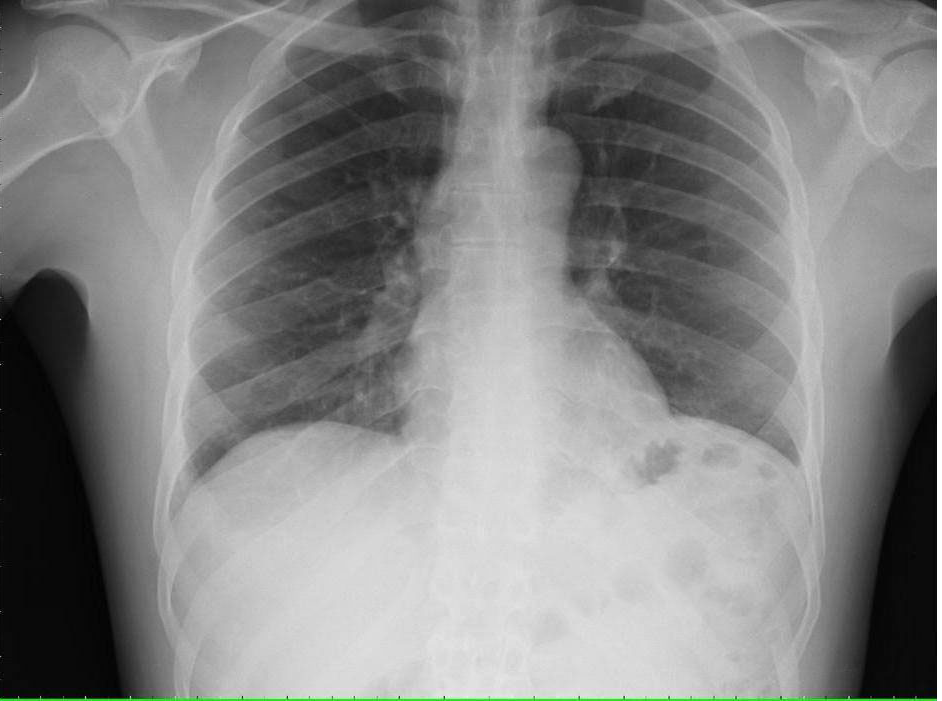
**Chest x-ray showing no evidence of active pulmonary disease**.

**Figure 2:**
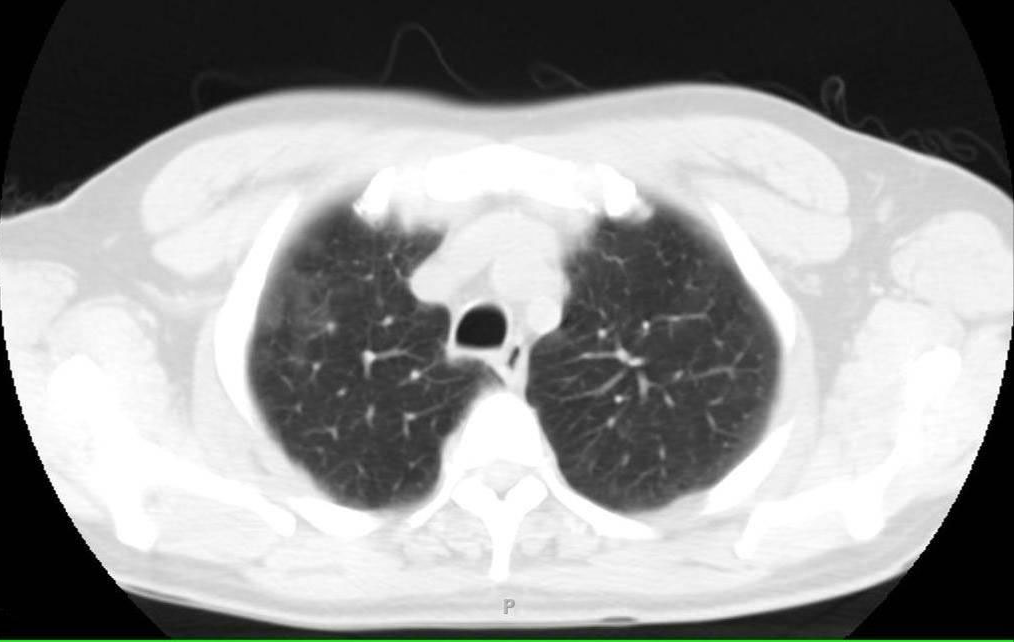
**CT scan showing subtle right middle lobe and right upper lobe ground-glass opacity**.

Laboratory tests revealed the following concentrations: sodium 138 mmol/L, potassium 4.5 mmol/L, chloride 103 mmol/L, bicarbonate 26 mmol/L, BUN 2.9 mmol/L, creatinine 61 μmol/L, glucose 4.78 mmol/L, white blood cell 3.4 × 10^9^/L, neutrophils 86%, hemoglobin 7.1 mmol/L, hematocrit 33%, platelets 322 × 10^9^/L, LDH 497 U/L, CD4+ lymphocyte count of 2. Chest X-ray was negative for infiltrate (Figure 1), but high resolution CT of the chest revealed subtle right middle lobe and right upper lobe ground-glass opacity (Figure 2).

The patient was admitted to the medical ward with a working diagnosis of pneumocystis pneumonia. A sputum sample was collected spontaneously for a culture work-up (Bartlett score: +3). No sputum sample was collected for mycobacterial stains. Empiric treatment with trimethoprim-sulfamethoxazole (1 tablet double-strength × 3 per day) and prednisone (40 mg × 2 per day) was started. The patient was also placed on treatment for HSV with acyclovir (400 mg × 3 per day). The prednisone doses were reduced as part of initial therapy for pneumocystis pneumonia. On the third day, direct fluorescent antibody test for *Pneumocystis jiroveci *was negative, but the culture was positive for *Bordetella bronchiseptica*. The patient was placed on droplet precautions; treatment was switched to levofloxacin (400 mg per day). Trimethoprim-sulfamethoxazole dose was modified to (1 tablet single-strength per day) for pneumocystis prophylaxis and azithromycin (1200 mg once per week) for *Mycobacterium avium complex *prophylaxis began. The patient subsequently improved on levofloxacin and on the seventh day was discharged home on levofloxacin, trimethoprim-sulfamethoxazole, azithromycin, and acyclovir. After eleven days, the patient visited our office. The patient had no complaints, no fever and no cough and his shortness of breath was fully resolved. On retrospect, the patient reported that he had been visited by his brother and his brother's dog at his home about a week prior to the illness.

## Discussion

Culture study of sputum revealed that the causative pathogen of pneumonia in our patient was *Bordetella bronchiseptica*. This is a pleomorphic Gram-negative coccobacillus which is a common respiratory tract pathogen in many domestic and wild animals which causes respiratory diseases in a broad variety of mammal species [4]. The route of transmission has not been clearly established. Occupational exposure to kenneled dogs in one case was documented [5]. A "kennel cough", also known as nonproductive paroxysmal cough, is a highly contagious form of tracheobronchitis in dogs [2]. *Bordetella bronchiseptica *has been also isolated as a human commensal, notably from the respiratory tract. There have been several cases reported in the literature in which *Bordetella bronchiseptica *was a causative agent of infection among humans. These range from acute iatrogenic maxillary sinusitis and tracheobronchitis to acute pneumonia and pneumonia with septicemia. This organism is typically a pathogen of immunocompromised hosts such as people who abuse alcohol, people with diabetes, patients with Hodgkin's disease or chronic lymphocytic leukemia and patients with HIV/AIDS [6].

Infection due to *Bordetella bronchiseptica *is uncommon in HIV-infected people. Respiratory illnesses ranged in severity from mild upper respiratory tract infection to pneumonia. There have been a few reports in the literature across decades, describing pneumonias in immunocompromised HIV-infected patients caused by *Bordetella bronchiseptica*. Medical records of over 41,000 patients with HIV researched by Dworkin *et al. *revealed that nine of the patients had been diagnosed with a *Bordetella bronchiseptica *infection during 1991-1998. Five patients suffered from pneumonia but *Bordetella Bronchiseptica *was the only pathogen isolated from a culture specimen in only two of the patients.

Presence of the pathogen was confirmed by specimen culture obtained by nasal swab, sputum or bronchoscopy. All patients had a CD4+ lymphocyte count less than 200. Most of these infections were treated successfully based on the antibiotic susceptibility pattern [5], although this pathogen when unrecognized and treated improperly can be fatal [7].

## Conclusion

Our case report points out the importance of a thorough culture work-up when caring for immunocompromised patients with HIV. The report also stress the importance of eliciting data of animal exposure. Physicians should recommend avoidance of unknown animals, as well as proper health care and vaccination of pets. We believe that *Bordetella bronchiseptica*, because of its similar presentation to pneumocystis pneumonia, should be considered in the differential diagnosis of pneumonia in people with HIV.

## List of abbreviations

AIDS: acquired immunodeficiency syndrome; BUN: blood urea nitrogen; CD4: cluster of differentiation type 4; CT: computed tomography; HSV-2: h type 2; LDH: lactate dehydrogenase.

## Competing interests

The authors declare that they have no competing interests.

## Authors' contributions

MG and JP analyzed, interpreted the patient's data, introduced and adjusted treatment, followed up during a hospitalization. JP continued the follow-up in outpatient setting. All authors read and approved the final manuscript.

## Consent

Written informed consent was obtained from the patient for publication of this case report and any accompanying images. A copy of the written consent is available for review by the Editor-in-Chief of this journal.
